# Usefulness of a stool to stabilize dental chairs for cardiopulmonary resuscitation (CPR)

**DOI:** 10.1186/s12873-019-0258-x

**Published:** 2019-08-08

**Authors:** Norimasa Awata, Takashi Hitosugi, Yoichiro Miki, Masanori Tsukamoto, Yoshifumi Kawakubo, Takeshi Yokoyama

**Affiliations:** 1 Department of Dental Anesthesiology, Faculty of Dental Science, Kyushu University, 3-1-1 Maidashi, Higashi-ku, Fukuoka, 812-8582 Japan; 2 School of Interdisciplinary Science and Innovation, Faculty of Arts and Science, Kyushu University, 744 Motooka Nishi-ku, Fukuoka, 819-0395 Japan; 3 Department of Dental Anesthesiology, Kyushu University Hospital, 3-1-1 Maidashi, Higashi-ku, Fukuoka, 812-8582 Japan

**Keywords:** Cardiopulmonary resuscitation (CPR), Manual chest compression (MCC), Dental chair, Stool, Dental surgery

## Abstract

**Background:**

Cardiopulmonary resuscitation (CPR) requires immediate start of manual chest compression (MCC) and defibrillation as soon as possible. During dental surgery, CPR could be started in the dental chair considering difficulty to move the patient from the dental chair to the floor. However, all types of dental chairs are not stable for MCC. We previously developed a procedure to stabilize a dental chair by using a stool. EUROPEAN RESUSCITATION COUNCIL (ERC) guideline 2015 adopted our procedure when cardiac arrest during dental surgery. The objective of this study was to verify the efficacy of a stool as a stabilizer in different types of dental chairs.

**Methods:**

Three health care providers participated in this study, and 8 kinds of dental chairs were examined. MCC were performed on a manikin that was laid on the backrest of a dental chair. A stool was placed under the backrest to stabilize the dental chair. The vertical displacement of the backrest by MCC was recorded by a camcorder and measured by millimeter. Next, the vertical displacement of the backrest by MCC were compared between with and without a stool.

**Results:**

In all 8 dental chairs, the method by using a stool significantly reduced the vertical displacements of the backrest by during MCC. The reduction ratio (mean [interquartile range]) varied between nearly 27 [20] and 87 [5] %. In the largest stabilization case, the displacement was 3.5 [0.5] mm with a stool versus 26 [5.5] mm without a stool (*p* <  0.001).

**Conclusions:**

Our procedure to stabilize dental chairs by using a stool reduced the displacement of a backrest against MCC in all chairs.

**Clinical relevance:**

Effective MCC could be performed in dental chairs by using a stool when sudden cardiac arrest occurs during dental surgery.

## Background

The dental office poses special circumstance where life-threatening emergencies of aspiration of dental materials and asphyxia can lead sudden cardiac arrest. We have already proposed supine abdominal thrust as a relief for asphyxia in the dental chair [1]. When the thrust relief is ineffective, immediate cardiac arrest can occur. Or cardiac arrest might occur alone, as dental surgery is often stressful for patients and dental surgery sometimes worsens basic illness. CPR requires immediate start of manual cardiac compression (MCC). The patient must be placed on a hard surface to ensure the effectiveness of MCC. However, given the limited space around a dental chair for effective interventions on the floor and the difficulty in moving a patient to the floor safely requiring multiple staff which may be limited in some clinics, CPR should be started in the dental chair itself. But, all types of dental chair are not always stable for MCC, because there is no steady support between the backboard of the dental chair and the floor. These condition may alter the effectiveness of MCC.

We previously reported the usefulness to stabilize a dental chair by using a stool for effective chest compression [2]. This procedure was adopted in the ERC guideline 2015 [3]. A stool is placed under the tilted or horizontal backrest, and then the dental chair is lowered so that the backrest come into contact with the stool to support the backrest of the dental chair. To our knowledge, however, there are many kinds of dental chairs, and the shapes of their backrest are different. In addition, the dental chairs have different seat-padding. Thus, it is unclear if our procedure is effective in all types of dental chairs.

The objective of this study was to evaluate the efficacy of a stool as a stabilizer for effective MCC. We compared the performance of MCC in different types of dental chairs with and without a stool. We hypothesized that a stool as a stabilizer may reduce the vertical displacement by MCC and increase the efficacy of MCC in dental chairs when the same quality of MCC is provided.

## Methods

### Study design and setting

Eight different dental chairs were used in this study. #1 (EOM-PLUS SS®; GC, Tokyo, Japan), #2 (EOM ∑®; GC, Tokyo, Japan), #3 (EOMαII®; GC, Tokyo, Japan), #4 (Celeb BM Type Clair®; TAKARA, Tokyo, Japan), #5 (SPACELINE EMCIA Type II®; MORITA, Tokyo, Japan), #6 (SPACELINE EMCIA Type III UP®; MORITA, Tokyo, Japan), #7 (NOVA SERIO®; YOSHIDA, Tokyo, Japan), #8 (STAGE II®; YOSHIDA, Tokyo, Japan). Each dental chair was installed in four private dental offices. Three health care providers, who completed AHA-certified Basic Life Support course, participated in this study; A: 47 years-old man,175 cm, 93 kg. B: 44 years-old man, 177 cm, 60 kg. C: 44 years-old woman, 157 cm, 50 kg.

**Fig. 1:**
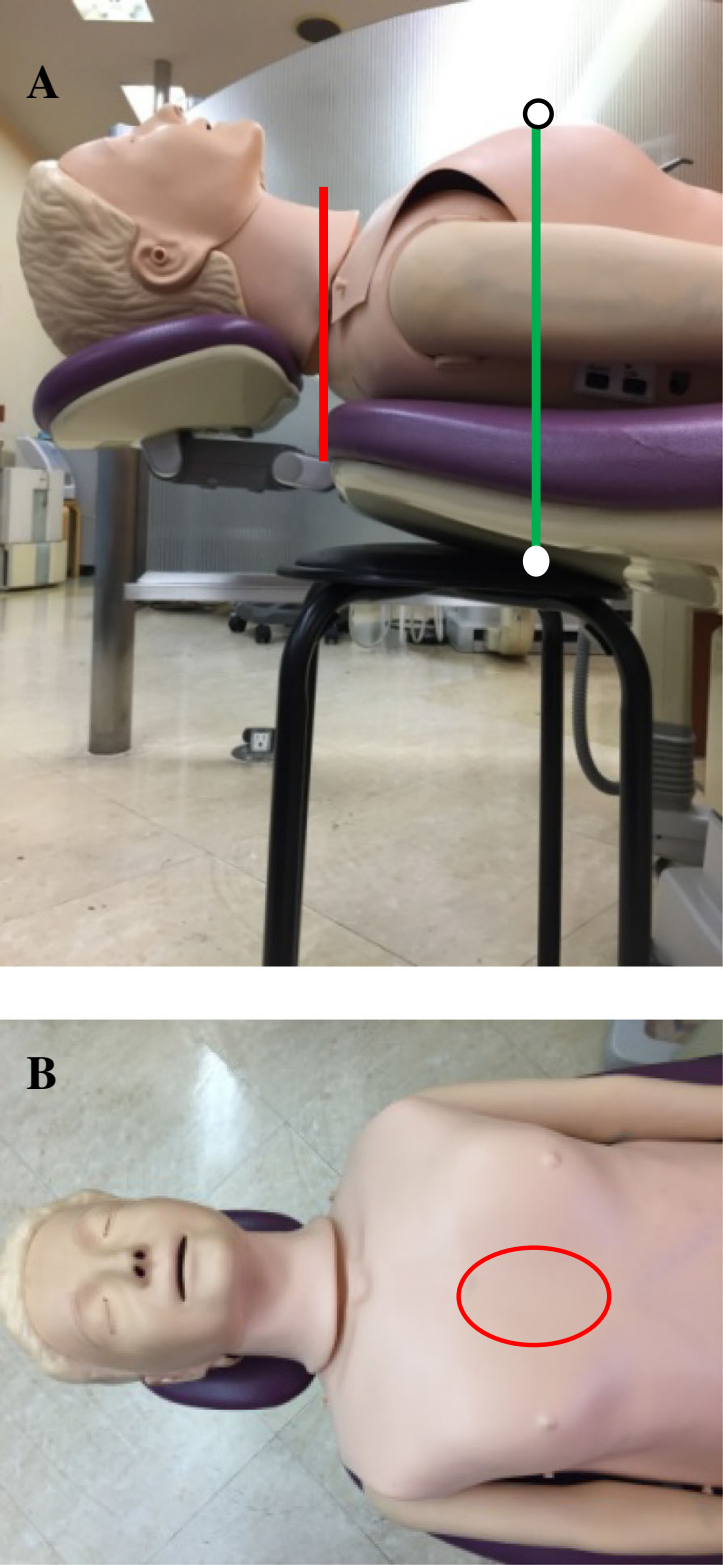
Setup of the manikin for measuring chest compression depth and movement of the backrest. Placement of the round stool as a stabilizer. The edge of the seating surface of the round stool was set to touch the backrest vertically under the area or chest compressions. **a** The hand position for the chest compressions was a center of the manikin’s chest (**b** Red ellipse)

The CPR manikin (Resusci Anne Torso Basic version 2011; Laerdal Medical AS, Stavanger, Norway) was laid on the horizontal backrest of the dental chair. The upper end of the torso of the manikin was aligned with the top edge of the backrest (Fig. 1a, Red line). The surface of the backrest under the lower half of the sternum of the manikin was levelled using a levelling instrument (Z-340; Hozan Co., Osaka, Japan).

**Fig. 2:**
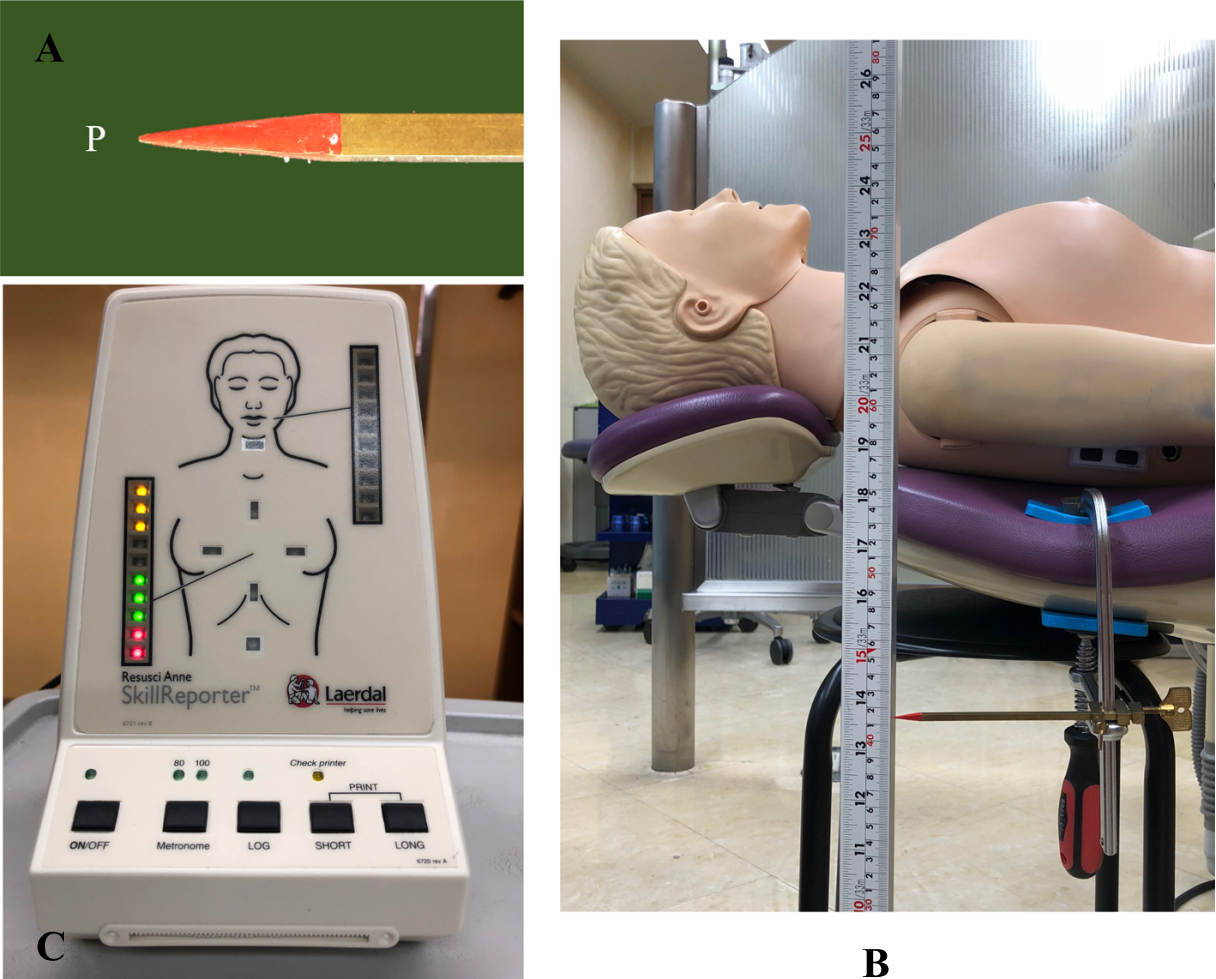
The displacement of the point P on the lower surface of the backrest (vertically under the area for external cardiac compression) was fixed a vertical-measurement instrument. The instrument was attached a metal indicator. **a**, **b** Chest compression depth was measured by the measurement equipment (skill-reporter®). The equipment glow green when chest compression depth was 3.8 to 5.0 cm, and red when 5.1 to 6.0 cm (**c**)

The hand position for MCC was the center of the chest (the lower half of the sternum, Fig. 1b) as recommended in the European Resuscitation Council Guidelines for Resuscitation [4] and the American Heart Association (AHA) Guidelines [5]. Three health care providers performed MCC on the resuscitation manikin in eight different dental chairs. The displacement of the point P (Fig. 2a) on the lower surface of the backrest (vertically under the area for MCC) was fixed (Fig. 2b). The metal indicator (point P) was attached the instrument by using a level gauge (Z-340; Hozan Co., Osaka, Japan) horizontally to the ground. The point P was measured at the same time as MCC-induced vertical movements of the backrest. The depth of MCC was kept between 5.1 to 6.0 cm with and without a stool. The actual depth of MCC was evaluated by the skill-reporter® system equipped with the manikin. The green light of the skill-reporter® indicates 3.8 to 5.0 cm of MCC depth, and red light indicates 5.1 to 6.0 cm of MCC depth (Fig. 2c). When the compression depth in the chest of manikin by MCC was 5.1 to 6.0 cm, the vertical displacements of the backrest from its basal position (the width of a starting point to an ending point) were recorded by the camcorder (HC-W580 M; Panasonic, Osaka, Japan). Video data were transferred to a computer (Dell; Windows 7, intel: Core i3, Cupertino CA, USA) using a camcorder’s dedicated software (HD Writer 3.1; Panasonic, Osaka, Japan). the vertical displacements (degree of instability) of the backrest were measured using the simultaneously captured ruler as a reference.

To compare the efficacy of a stool as a stabilizer on MCC in eight types of dental chair, a round stool with a hard seating surface (45 cm in diameter, 46 cm in height; FB-01ALLBK, Fuji Boeki Co., Ltd. Fukuoka, Japan) was placed under the backrest of the dental chair. The edge of the seating surface of the round stool was set to (vertically) touch the backrest under the area for ECC (Fig. 1a, Green line). MCC was performed with or without the round stool as a stabilizer. The manikin was on a fully reclined chair.

### Protocol

Three health care providers individually performed 10 rounds of continuous MCC for 20 times each at a pace of 100 compressions per minute by synchronizing to a metronome. Chest compressions of 5.1 to 6.0 cm were performed with and without a stool. The health care providers and the research team were blinded to the information during MCC. Therefore, for each participant, 200 records of chest compressions were gotten for each dental chair.

### Statistical analysis

The programming language R (version 3.4.3; The Comprehensive R Archive Network, USA) was used for statistical analysis.

Each combined measurement data set of the chair’s reference point displacement during ECC treatment by 3 practitioners were applied to the Shapiro-Wilk test (with the function shapiro.test) to see whether they were sampled from a population with the normal distribution. The data sets were analyzed using the non-parametric Wilcoxon rank sum test (wilcox.exact: exactRankTests package).

## Results

**Fig. 3:**
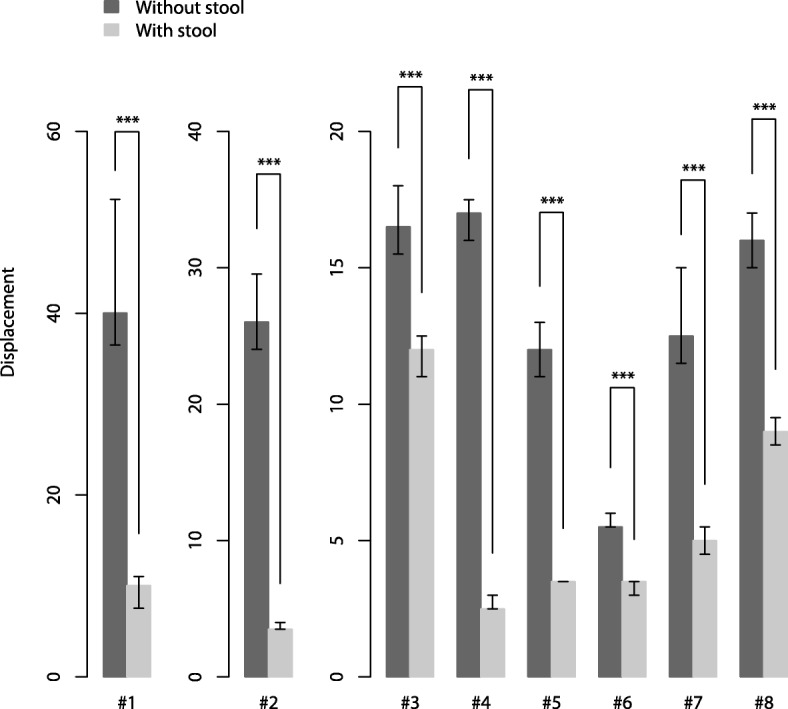
Effect of the stool (stabilizer) on the vertical movements of the backrest caused by external cardiac compression. Results are expressed as median [interquartile range]. Asterisks represent significant differences (***P <  0.001), (#: Number of Dental chair)

The vertical displacements of the backrest of the dental chair induced by MCC were investigated with or without a round stool as a stabilizer. Four thousand eight hundred times of MCC was recorded, but 34 of them was excluded as inappropriate compression or unclear recording. The stool which placed under the backrest as a stabilizer significantly reduced the vertical displacements of the backrest in all eight dental chairs (Table. 1, Fig. 3). For example, the largest stabilization (87 [5] %) was typically observed in Chair #2, where the displacement of the backrest by MCC was 3.5 [0.5] mm with the stool, while that was 26 [5.5] mm without one. Eq. 1 defines the reduction ratio.

1 $$ \mathrm{reduction}\ \mathrm{ratio}=1-\frac{\ \mathrm{displacement}\ \mathrm{with}\ \mathrm{stool}}{\ \mathrm{displacement}\ \mathrm{with}\mathrm{out}\ \mathrm{stool}} $$

**Table 1:** Effect of the stool (stabilizer) on the vertical movements of the backrest caused by MCC. Results are expressed as median [interquartile range]

Chair	Displacement without stool [mm]	Displacement with stool [mm]	Reduction ratio [%]	*p*-value
#1	40 [16]	10 [3.5]	75 [19]	< 0.001
#2	26 [5.5]	3.5 [0.5]	87 [5]	< 0.001
#3	16.5 [2.5]	12 [1.5]	27 [20]	< 0.001
#4	17 [1.5]	2.5 [0.5]	85 [4]	< 0.001
#5	12 [2]	3.5 [0]	71 [5]	< 0.001
#6	5.5 [0.5]	3.5 [0.5]	36 [15]	< 0.001
#7	12.5 [3.5]	5 [1]	60 [19]	< 0.001
#8	16 [2]	9 [1]	44 [14]	< 0.001

(#: Number of Dental chair) #1 (EOM-PLUS SS®; GC, Tokyo, Japan), #2 (EOM ∑®; GC, Tokyo, Japan), #3 (EOMαII®; GC, Tokyo, Japan), #4 (Celeb BM Type Clair®; TAKARA, Tokyo, Japan), #5 (SPACELINE EMCIA Type II®; MORITA, Tokyo, Japan), #6 (SPACELINE EMCIA Type III UP®; MORITA, Tokyo, Japan), #7 (NOVA SERIO®; YOSHIDA, Tokyo, Japan), #8 (STAGE II®; YOSHIDA, Tokyo, Japan)

The reduction ratios were between nearly 27 [20] ~ 87 [5] % and different by chairs (Fig. 3). The support efficiency of a stool ware different, maybe as the backrest of some dental chairs has an outer-shell shape with curving line. #2 and #8 dental chairs have a flat outer-shape relatively. Therefore, the stool supported the backrest of dental chair firmly (Fig. 5a). In this situation, about 87% decrease in the displacement of a backrest against MCC by the stool. In #1 dental chair, the shape has a steep curve. Consequently, the stool contacted to the backrest at a smaller point where the MCC’s force concentrates on (Fig. 5b). In these situations, the reduction rate is smaller than that of #2 (87 [5] %), although, it also significantly decreased the vertical displacement by MCC.

## Discussion

The efficacy of a stool as a stabilizer for CPR was investigated in 8 kinds of dental chairs in this study. To our knowledge, this is the first report to compare the stability in many types of dental chairs using our method. This study showed that the stool significantly reduced the vertical displacement of the backrest against MCC. All health care providers could perform stable ECC in all chairs with a stool.

ERC and AHA, current guidelines emphasize the importance of pushing hard and fast, and of minimizing interruptions during compression [4, 5]. MCC should be started on the stable surface as soon as possible when cardiac arrest was suspected. During CPR in the dental chair, however, backrest of dental chairs may be not firmly supported for ECC. Previously, we developed a method to stabilize a backrest of a dental chair by using a stool [6]. This method has been adopted in the ERC Guideline 2015 [3]. However, there are many types of dental chairs in the world, and these chairs equip different types of backrest, cushion, pad-softness and a hinge (joint) between the backrest and the seat. It was not clear whether MCC would be performed effectively or not on every types of dental chair. Therefore, we investigated the method of a stool as a stabilizer [2, 6]. In CPR, a large vertical displacement of the backrest might decrease efficacy of MCC [7]. In addition, the vertical displacement increases labor efforts as additional power to push down is required. In these situation, ECC may cause more fatigue especially for rescuers in the light body weights group [8–10].

**Fig. 4:**
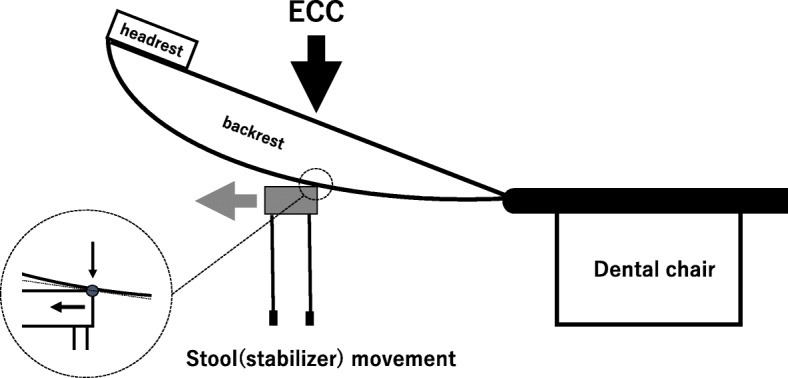
A position of a stool as a stabilizer with a dental chair for MCC

**Fig. 5:**
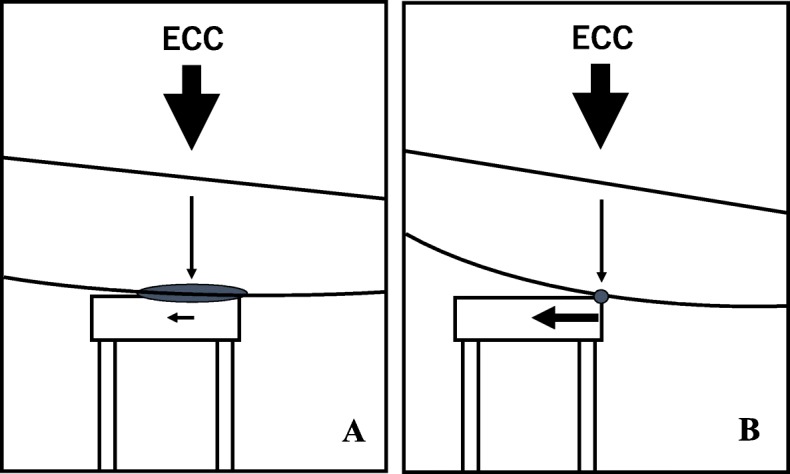
A contact area of a stool with a backrest of a dental chair. An outer-shell shape of backrest has curving line, a contact area gets narrow. Power of ECC concentrate on the area. A stool could not sustain the power and moves laterally

In this study, our method [2, 6] significantly reduced the displacement of a backrest against MCC in all dental chairs. During MCC in the dental chair, the displacement of the backrest seemed to be mainly caused by stool movements and cushions or seat-pads-softness. A stool moved just a little in every MCC (Fig. 4). The support efficiency of a stool was different, maybe as the backrest of some dental chairs has a curved outer-shell. #2 and #8 dental chairs have a flat outer-shape relatively. Therefore, the stool supported the backrest of dental chair firmly (Fig. 5a). In this situation, about 87% decrease in the displacement of a backrest against MCC by the stool. In #1 dental chair, the shape has a steep curve. Consequently, the stool contacted to the backrest at a smaller point where the MCC’s force concentrates on (Fig. 5b). In these situations, the reduction rate is smaller than that of #2 (87 [5] %), although, it also significantly reduced the vertical displacement by MCC.

Limitations of the study should be mentioned. First, this study includes only 8 kinds of chairs. These are common models, although there are a lot of typical chairs in Japan. Second, this study was performed on a manikin model, which cannot be extrapolated to a human faithfully. Third, this study did not consider the effect of the cushion-pad of the backrest. Next, the stool was set a particular position where was just under the area for MCC. Further studies should be conducted to evaluate other position of the stabilizer where is more effectively. And, the usefulness of other types of stabilizers remains to be verified. However, no studies to date have demonstrated a significant reduction in deflective movements on several types of a dental chair. The technique is very easily and helpful method, and must use at time of CPR on a dental chair.

## Conclusion

Our method could significantly reduce the vertical displacement of dental chairs by MCC, and it is convenient and useful when sudden CPR is required. We have only to recline the backrest to horizontal position, place the stool below the back rest and down the chair to contact with the stool firmly.

## Data Availability

The data sets during and/or analysed during the current study available from the corresponding author on reasonable request.

## Abbreviations

AHA: American Heart Association
CPR: Cardiopulmonary resuscitation
ERC: EUROPEAN RESUSCITATION COUNCIL
MCC: Manual chest compression
